# Reinforce Bee Product Quality Evaluation to Protect Human Health

**DOI:** 10.3390/foods12224143

**Published:** 2023-11-16

**Authors:** Qiangqiang Li, Liming Wu

**Affiliations:** State Key Laboratory of Resource Insects, Institute of Apicultural Research, Chinese Academy of Agricultural Sciences (CAAS), Beijing 100093, China; liqiangqiang@caas.cn

## 1. Introduction

The quality of bee products is directly related to the health of consumers. Ensuring that bee products meet the required standards is essential to improve their quality. Osaili et al. conducted a study examining the physicochemical characteristics of honey imported into the United Arab Emirates (UAE) through Dubai ports from 2017 to 2021, analyzing 1330 samples for sugar components, moisture, hydroxymethylfurfural (HMF) content, free acidity, and diastase number [1]. The results showed that while 79.2% of honey samples met Emirates honey standard requirements, 20.8% did not comply due to possible adulteration or improper storage or heat treatment. Raweh et al.’s investigation into local and imported honey samples revealed significant differences in their physicochemical properties such as moisture level, color, electrical conductivity (EC), free acidity (FA), pH value, diastase activity, HMF content and individual sugar content [2]. These studies emphasize the importance of comprehensive analysis of physicochemical parameters for standardizing bee products and increasing awareness regarding quality investigations.

## 2. Honey Quality Evaluation

The identification of honey adulteration with various sugar syrups poses a complex and challenging issue, which is crucial for ensuring equitable trade and protecting the interests of beekeepers. Yan et al. developed an efficient approach to detect adulterants in honey by employing fluorescence spectroscopy to analyze the emission spectra and frequency doubled peak (FDP) intensity at 740 nm, which exhibit distinguishable characteristics between honey and sugar syrups [3]. Furthermore, Hao et al. utilized fluorescence spectroscopy combined with chemometrics to investigate a rapid and non-destructive method for identifying corn sugar syrup as an adulterant in wolfberry honey [4].

Additionally, the authentication of honey is crucial for ensuring quality control. Żak et al. collected data on various techniques used to evaluate the quality and authenticity of honey, while also highlighting the challenges encountered during this evaluation process [5]. Furthermore, Dżugan et al. utilized SDS-PAGE and HPTLC techniques to identify distinctive protein and polyphenolic profiles as a means of authenticating goldenrod honey [6]. Sęk et al. conducted a study focusing on two important factors in honey quality control: the diastase number (DN) and HMF content, alongside melissopalynological analysis of manuka honey. The investigation revealed significant fluctuations in the percentage of *Leptospermum scoparium* pollen found in manuka honey. Moreover, a considerable proportion of manuka honey exhibited low DN levels [7].

The storage conditions also influence the quality of honey. Živkov Baloš et al. conducted a study to evaluate the quality and stability of sunflower honey during storage by analyzing its physicochemical parameters such as water content, HMF content, diastase activity, pH value, and free acidity [8]. The findings revealed that after being stored at room temperature for 18 months, there was a significant decrease in diastase activity but a notable increase in HMF content. Moreover, the pH value of honey decreased from 3.66 to 3.56, despite relatively stable levels of water content and free acidity.

## 3. Bee Pollen Quality Evaluation

The nutritional significance of bee pollen is paramount, as it encompasses a diverse range of bioactivities that contribute to disease prevention. Varied species of bee pollen possess distinct nutrient profiles and bioactive compounds, thereby exhibiting differential bioactivity. Oroian et al. conducted a comprehensive analysis of various parameters including organic acids, total phenols, total flavonoids, individual phenolic compounds, fatty acids and amino acids in Romanian bee pollen [9]. Additionally, Rojo et al. assessed the differences in levels of total phenols and flavonoids as well as antioxidant activity among bee pollen samples sourced from different botanical origins within Galicia, northwest Spain [10]. Qi et al. identified L-theanine (864.83–2204.26 mg/kg) and epicatechin gallate (94.08–401.82 mg/kg) as exclusive markers for the quality evaluation of *Camellia* bee pollen [11]. The results demonstrated the extensive botanical diversity and nutrient richness of bee pollen, thereby enhancing its potential as a valuable nutritional supplement.

However, there is a concern regarding the safety of bee pollen due to its potential to trigger food-related allergies. Tao et al. conducted a study employing cellulase, pectinase, and papain for the hydrolysis of allergens in bee pollen and investigated the impact of enzyme treatment on allergic reactions in BALB/c mice [12]. The results demonstrated that enzyme-treated bee pollen effectively reduced scratching frequency in mice, mitigated tissue damage caused by allergies, decreased serum IgE levels, and regulated bioamine production. Furthermore, it was observed that enzyme-treated bee pollen influenced metabolic pathways and modulated gut microbiota composition in mice. These findings indicate that enzyme-treated bee pollen has the potential to be utilized as a hypoallergenic product for consumer consumption.

## 4. Royal Jelly Quality Evaluation

Royal jelly is highly valued by consumers for its health benefits attributed to its abundant protein, peptides, lipids, and other bioactive nutrients. Consequently, the quality assessment of royal jelly plays a pivotal role in ensuring the market’s sustainable and healthy development. Considering that the lipid content in royal jelly originates from bee-collected pollen, and different plant sources of pollen can influence the lipid composition of royal jelly, Zhou et al. conducted an investigation to explore how feeding honeybees with various types of pollen affects the lipid composition of royal jelly [13]. The findings unveiled substantial variations in the phospholipid and fatty acid content in royal jelly produced by honeybees fed with pollen containing diverse lipid components, thereby exerting an impact on its overall quality. This research offers valuable guidance for producing premium-grade royal jelly.

## 5. Pesticide Detection in Bee Products

Honeybees are exposed to a significant amount of environmental pollutants during their collection of nectar and pollen from plants. Consequently, upon entering the beehive, a substantial portion of these pollutants is inevitably transferd to bee products. Kasiotis et al. employed two effective multi-residue analysis methods, namely HPLC-ESI-MS/MS and GC-MS/MS, to ascertain the presence of more than 130 pesticides and their metabolites in 109 honey and bee pollen samples collected between 2015 and 2020 [14]. The findings revealed that the pesticides detected in honey and pollen with higher residue levels primarily comprised coumaphos, imidacloprid, acetamiprid, amitraz metabolites (DMF and DMPF), as well as tau-fluvalinate. Therefore, it is imperative to conduct pesticide residue detection and risk assessment in bee products for the preservation of human health.

Finally, we express our sincere gratitude to the authors who have made significant contributions to this volume through their unwavering dedication and exceptional expertise. We cordially invite readers of this article to provide valuable feedback and constructive suggestions on the content under consideration, including any potential typographical errors.
